# A qualitative analysis of transitions to heroin injection in Kenya: implications for HIV prevention and harm reduction

**DOI:** 10.1186/s12954-015-0061-2

**Published:** 2015-09-04

**Authors:** Andy Guise, Margarita Dimova, James Ndimbii, Phil Clark, Tim Rhodes

**Affiliations:** Centre for Research on Drugs and Health Behaviour, London School of Hygiene and Tropical Medicine, 15-17 Tavistock Place, London, WC1H 9SH UK; School of Oriental and African Studies, Thornhaugh Street, London, WC1H 0XG UK; Kenyan AIDS NGOs Consortium, Jabavu Lane, P.O. Box 69866–00400, Nairobi, Kenya

**Keywords:** Transitions, Initiation, Smoking, Heroin, Injecting drug use, HIV, Harm reduction, Kenya, Africa, Structure

## Abstract

**Background:**

Heroin injection is emerging as a significant dimension of the HIV epidemic in Kenya. Preventing transitions to injecting drug use from less harmful forms of use, such as smoking, is a potentially important focus for HIV prevention. There is, however, little evidence to support comprehensive programming in this area, linked to a shortage of analysis of the social and structural context for transitions, particularly in low-income settings. We explore accounts of transitions from smoking to injecting in Kenya to understand the role of individual, social and structural processes.

**Methods:**

We combine data from two separate studies conducted in Kenya: an in-depth qualitative study of HIV care access for people who inject drugs (study 1) and an ethnographic study of the political economy of the heroin trade in Kenya (study 2). In-depth interviews with PWID and community observation from study 1 are triangulated with accounts from stakeholders involved in the heroin trade and documentary data from study 2.

**Results:**

People who inject drugs link transitions to injecting from smoking to a range of social and behavioural factors, as well as particular aspects of the local drug supply and economy. We present these results in the form of two narratives that account for factors shaping transitions. A dominant narrative of ‘managing markets and maintaining a high’ results from a process of trying to manage poverty and a shifting heroin supply, in the context of deepening addiction to heroin. A secondary narrative focuses on people’s curiosity for the ‘feeling’ of injecting, and the potential pleasure from it, with less emphasis on structural circumstances.

**Conclusions:**

The narratives we describe represent pathways through which structural and social factors interact with individual experiences of addiction to increase the risk of transitions to injecting. In response, HIV and harm reduction programmes need combinations of different strategies to respond to varied experiences of transitions. These strategies should include, alongside behaviour-oriented interventions, structural interventions to address economic vulnerability and the policing of the drug supply.

## Background

Increasing numbers of people in Kenya are injecting drugs, with estimates suggesting there are 18,000 people injecting heroin, with a human immunodeficiency virus (HIV) prevalence amongst this population of 18 % for men and 44 % for women [1]. There are also reportedly high numbers of people smoking heroin, in a ‘cocktail’ where heroin is mixed with tobacco and ‘bhangi’ (cannabis); anecdotal reports suggest there are many more people smoking than injecting heroin. Whilst all methods for using heroin can be linked with harms, injecting is considered the most dangerous through its links to multiple health and social harms [2], including HIV, hepatitis C, skin and vein damage and arrest and imprisonment. The Kenyan government with civil society partners has recently introduced harm reduction services for people who inject drugs (PWID), including needle and syringe exchange and opioid substitution therapy. However, there are limited strategies available to support those who smoke heroin, but do not inject, including limited access to residential rehabilitation and methadone substitution. Support for people smoking heroin, and in particular supporting the prevention of transitions to injecting drug use, is therefore a potentially key priority for HIV prevention. In this paper, we combine insights from two research studies in Kenya to address key gaps in the literature: transitions in low- and middle-income countries and then the role of social and structural factors in these.

The majority of research on transitions delineates individual behavioural factors and then interpersonal- or social-level factors [3, 4]. Such work draws attention to how injecting becomes acceptable, even appealing, through social interaction [5] and how injecting is demonstrated and inspired by social contacts [6, 7]. Overlapping with this is a concern for curiosity and pleasure [5, 7] or ‘the rush’ of injecting as compared to other modes of use [8]. Alongside pleasure is a concern to inject so as to use heroin more economically [6, 7]. This literature has also drawn attention to the potentially greater vulnerability of women to transitions [9, 10] and of younger people [7]. Transitions can, however, perhaps be most usefully understood as shaped by a ‘constellation’ of factors from the personal, socio-economic and political spheres [6, 11]. The role of macro-level, structural factors such as the role of drug supply shifts and policing [12–14] but also the political-economic context and impact of social and economic change [15, 16] is less of a focus in current literature. Research in low- and middle-income settings is also limited [6, 7, 12, 15].

Available interventions reflect this focus of research by being oriented towards addressing individual behaviour and discouraging peer influence within social networks [2, 17, 18]. Whilst such interventions are potentially valuable, in isolation, they risk ignoring the role of the social and structural environment in producing increased risk for transitions. There is now a rich tradition of research highlighting the role of the structural and social environment in creating risk for harm from drugs, as critique of analysis focusing solely on individual and behavioural determinants of harm [11, 19, 20]_._ The risk of harm from practices such as injecting drugs are, from this perspective, shaped by interactions between individuals and their environment, where structural factors constrain and enable different choices [19]. Transitions to injecting can therefore be understood as shaped by how expectations and understandings of injecting drug use, stigma, economic pressures and other factors with influence emerge from an individual’s experience of particular social conditions.

An exploration of social and structural influences on transitions from smoking to injecting heroin in Kenya emerged from discussion of initial findings from a qualitative longitudinal study of HIV care access (the Access2Care study; Kenya AIDS NGOs Consortium, London School of Hygiene and Tropical Medicine and International HIV/AIDS Alliance). Through early phases of the study, people who inject drugs spoke about the role of friends, poverty, shifts in the local tourist economy and shifts in the drug supply as key influences in transitioning from smoking to injecting heroin. The Access2Care study was implemented at the same time as the Heroin Trade study. Discussions between the two teams indicated the value in combining analyses to explore in detail how structural processes, such as an evolving drug supply and the policing of it, impact on transitions. Context for the development of these themes of enquiry was past research in Kenya reporting a major shift in the heroin supply from ‘brown sugar’ to ‘white crest’ heroin in the early 2000s, which in turn led to a widespread shift from chasing to injecting [12, 21] and more recent reports of interruptions to the drug supply influencing transitions [22]. Through conversations across the research teams facilitated by the London International Development Centre, we identified an opportunity to triangulate our findings and explore the contexts that produce increased risk for transitions further. In the rest of this paper, we report key themes from the Access2Care study in PWID accounts of their transitions and then triangulate this with detailed analysis of the drug supply from the Heroin Trade study. Understanding the role of these social and structural factors in shaping drug use is an urgent public health priority and could inform harm reduction strategies in Kenya and similar settings across the region.

## Methods

This paper combines data from two independent studies: the Access2Care (A2C) study (TR, JN, AG) and the Heroin Trade study (MD, PC). Both studies had an iterative research design, to allow in-depth exploration of a focus area: HIV care access and the political economy of the heroin trade in Kenya. We combined insights through two steps: (i) consultation on the developing analysis of the A2C study to allow further exploration of emerging hypotheses within the ongoing implementation of that longitudinal study and (ii) triangulation of data in the final analysis.

The Access2Care study was a longitudinal qualitative study working with PWID in three sites across Kenya—Nairobi, Malindi and Ukunda—from Dec 2012 to June 2014. In-depth ethnographic PWID interviews, interviews with community stakeholders and observation in community settings explored experiences of HIV care. Within these interviews and observations, accounts of transitions were raised by respondents alongside exploration of HIV treatment and prevention, needle and syringe use, drug treatment and other issues. Across the study, we interviewed 118 individuals, including a purposive sample of 33 people who were interviewed over three waves (wave 1 was Dec 2012/Jan 2013, with follow-ups at 6 and 12 months). Interviews were done by TR, JN, AG and outreach workers trained in qualitative interviewing. Sampling purposively sought a range of experiences of HIV care and injecting drug use by both genders. Interviews were conducted in English or Kiswahili, depending on the language skills and preference of the interviewee. Interviews were shaped by context: we sought to respond to the issues raised by respondents, and interviews were done in community settings, sometimes drug-using sites, and were limited and often shortened by the contingencies of drug use and addiction: withdrawals, hunger or the threat of arrest. It was within this research design and context that transitions as shaped by the social and structural context emerged in respondents interviews. Fifty PWID offered accounts of their transitions from smoking to injecting heroin, varying from brief utterances of a single factor to extended multi-layered accounts. We focus analysis on these accounts, relating in turn to the views of other respondents and stakeholders in exploring the context for them. Study participant characteristics are summarised in Table 1.

**Table 1:** Summary of participant characteristics for the two studies

Access2Care		
PWID reporting transitions: *N* = 50	Age (mean) and range	31 (22–45)
	Gender	13 women (26 %), 37 men (74 %)
	HIV status	15 living with (30 %)
Of the overall sample of 118, there were 84 men (71 %)/32 women (29 %) and 44 PLHIV (37 %), with an age range of 31 (19–49)		
Stakeholders: *n* = 12	7 male, 5 female—2 outreach project managers, 5 outreach workers, 2 outreach-based clinical officers, 1 clinical-based clinical officer, 2 outreach-based HIV counsellors	
Heroin Trade study		
Drug merchants: *n* = 35	32 male, 3 female, all participating in the informal, criminal economy	
Stakeholders: *n =* 57	54 men, 3 women, from police, government departments, rehabilitation centres and community organisations	

The Heroin Trade study was conducted over 8 months in multiple heroin distribution and consumption locations in Nairobi and throughout the coast of Kenya (and so including Malindi and Ukunda as cited for the Access2Care study) in 2013 and 2014 (and so, at the same time as the A2C study) by MD and PC. It relied on ethnographic methods to document interpersonal relations amongst drug merchants, community members and law enforcers through the field researcher’s immersion in the everyday lives of these populations. This produced a total of 132 in-depth, semi-structured interviews with 92 respondents in Kiswahili. A summary of participants is included in Table 1. Participant observation and critical document analysis supplemented these interviews.

Each study separately followed principles of thematic analysis [23], with emergent themes the focus for discussion between the teams, as part of an ongoing iterative process of analysis. In the analysis presented, we use findings from the A2C study to give insight into PWID experiences and then critically explore them with reference to contextual data from the A2C and Heroin Trade studies. Both studies secured relevant ethical approval, with the Access2Care study approved by the University of Nairobi and the London School of Hygiene and Tropical Medicine, whilst the Heroin Trade study was approved by the University of London. All participants provided informed consent, and names used in the text are pseudonyms to protect anonymity.

## Results

In the A2C study, PWID narrated their transitions from smoking to injecting by referring to a range of factors or processes. For example:

“R: Sometimes I see them, they are ten new comers who come from smoking to inject […]You know why, like me, the way how I get injected, I smoked a lot and I don’t feel anything. But these drugs we are taking is not real heroin, it is just chemicals.....so people they see us, we inject, we feel good and don’t spend a lot of money.” (Sandra, PWID, Ukunda, A2C)

Sandra’s account was typical for how it weaved together a combination of influential factors at the individual (‘I don’t feel anything’), social (‘they see us, we inject’) and structural level (the drug supply is of low quality). Key themes across the A2C study accounts focused on the active role of friends in promoting injecting, deciding to inject and managing risk, the feeling, the quality and the cost. These themes then overlapped with accounts of the context: that of poverty for people using drugs in the A2C study and that of a shifting drug supply and how it is policed from the Heroin Trade study.

These overlapping themes combine around two principal narratives that account for transitions: first, that of ‘managing the market and maintaining a high’ and a second of ‘the feeling of injecting’. We identified these narratives from the 50 PWID who discussed their transitions in the Access2Care study. As demonstrated in Table 1, these participants were mostly male (37, 74 %/13 women, 26 %), with an average age of 31, and 15 from this group were living with HIV; these characteristics are similar to those of the overall study sample, and in line with the key trends in Kenya of principally being men who inject drugs, with a high prevalence of HIV across the population. We outline these two narratives here, exploring how individual experiences are grounded in the social context.

### Managing the market and maintaining a high

A dominant narrative across the A2C study—figuring across 29 of the 50 accounts—was of a transition to injecting grounded in consideration of the material circumstances of money and the drug supply. Reflecting how economic concerns are central to organising daily life, people spoke of wanting a high, to recover from withdrawals or a growing tolerance for heroin in terms of the efficiency of injecting and how to balance structural changes in the economy and drug supply. For many, injecting was valued over smoking for how it allowed you to ‘stay until evening without taking another sachet’ (Joseph, Nairobi), or the high lasted for 8 hours, rather than 4 hours for smoking. This focus on time and the length of a high—not its intensity—is a function of the punishing cycle of looking for money, which occupied much of people’s daily life. Rather than ‘slaving’ to get enough money to smoke you could inject (Pep, Nairobi) and so avoid the ‘slaving’, or ‘hustling’ for money that was time-intensive, physically demanding and often brought little money. As well as the duration of a high was how smoking heroin could be reconciled with the other costs of daily life in the context of money shortages:

“I: what made them to join the injection?

R: its depending on the amount of money one is getting. You know some are getting a thousand [shillings] per day [approx. $10]. So this one thousand…if they [the dealers] sell drugs like one hundred and fifty [shillings], four drugs are six hundred shillings, and in the evening he is required to pay a room with two hundred, because he cannot sleep outside, so in that one thousand if you remove six hundred for drugs, and two hundred for a room, you will remain with two hundred for food.” (Haj, Nairobi)

A daily process of seeking money to address first the need for drugs, then shelter and then food was difficult and created an economic pressure to inject. As Isaiah, from Ukunda, said, injecting is cheaper, you do not ‘waste’ money.

Overlapping with a concern for cost, PWID spoke about how shifts in the quality of available heroin were central in their transitions. Echoing the findings of Beckerleg et al. [12], the historical shift from brown sugar to white crest heroin was cited by some as structuring their transition. This abrupt historical shift in supply has now given way to more regular fluctuations in the quality:

“I: You were smoking, why did you start injecting?

R: Okay sometimes it’s with the drug you know. This drug in the market it doesn’t have that kick, you know, the kick you want, you see. Now you see, the only way you are going to use it is by injecting, that’s when you will feel it maybe. So you end up injecting yourself, you see.” (Charo, Nairobi)

PWID spoke of how the heroin they bought was mixed with other substances, such as caffeine, dust and even biscuits. More needed to be bought or a different method found for administration.

A transition often figured as a highly managed process. There were isolated accounts where people spoke of confusion and feeling out of control, but more were spoken of as active decisions based on considerations of benefits or circumstances, albeit under considerable pressure:

“R: I smoked and reluctantly I decided to start injecting’

I: maybe it is somebody who introduced you?

R: I wanted it myself

I: you wanted to start?

R: I felt reluctant and decided to inject because anytime I smoke I spend a lot of money.” (Naomi, Ukunda)

For some, transitions were presented as a solution to a particularly acute shortage of money and had only intended to inject for just 1 day. Friends who already injected, or others in the highly public drug-using scene, were also identified in this process of decision making: sometimes this was the passive example from others, but friends were also described as proactively advocating the cost benefits of injecting as compared to smoking and then also helping by administering the first injection.

The risk to health from injecting was sometimes acknowledged in the context of these transitions; some reported not being aware of HIV and other harms from injecting, but the majority expressed some awareness of the risks from needle sharing and then overdose. This risk of HIV was often managed: people would purposively buy or source a new needle and syringe, with friends and others injecting figuring centrally in this process. There were only isolated accounts of people spontaneously deciding to inject and using a used needle and syringe.

#### The context of economic hardship: a dangerous, marginal position

The experience of daily life and a transition as a series of calculations over the availability of money and resources emerges from drug users’ marginal position in local economies. Any work that is available is informal, uncertain and often linked to physical risk. Across the A2C study, people often spoke of money, employment and the challenges of everyday survival as their priority, rather than the risk of HIV or other health and social concerns. In the absence of formal or reliable sources of income—denied through a combination of not being trusted, not being physically able to work or not being able to organise work within the demands of addiction—people engaged in shifting combinations of informal work: from carrying luggage at markets or sex work to petty crime. Getting money often involved time-intensive and demanding work: ‘difficult, yeh, difficult, you have to toil, you really have to toil. Like today I woke up around 5:30 in the morning because I don’t want the rush hour to end before I get the money that I want’ (Charo, Nairobi). Getting money was often dangerous, linked to petty crime as a focus for many. The majority of those interviewed across the A2C sample had experienced ‘mob justice’: physical violence from the community in response to real or perceived crime. A reliance on crime or informal work reflects an overriding experience of economic isolation: most had limited contact with family and there were few, if any, economic safety sets.

The structures of local economies featured not just marginal work for people who used drugs but economic cycles that exacerbated vulnerability and created particular acute financial pressures. In Malindi and Ukunda, and for much of coastal Kenya, the economic cycle is dominated by seasonal variations of tourism. The low tourist season is a particularly difficult time of the year to get money and as a result was linked with people transitioning:

“I: is there a lot of other people who have just started injecting?

R: yeah, I believe, some, there are some, there is a big group who are doing secretly, because at the moment you see the climate it’s very rainy and at the beach there are no guests, so coming to get money it’s very hard.” (Victor, Ukunda)

Whether through formal work in hotels, selling souvenirs or sex or seeking donations, many were reliant on how the tourism industry would trickle down through the local economy. With pre-existing economic vulnerability, people were reportedly unable to manage these structural shifts.

#### The context for a shifting supply: insight from the Heroin Trade study

The concurrent Heroin Trade study provides detailed insight into the shifts in the quality of heroin PWID describe in the Access2Care study. Structural characteristics of the Kenyan drug supply shape the fluctuating cost and quality of heroin. More heavily policed—and thus diversified and fragmented—supply routes through and into East Africa reflect global illicit drug shipment patterns [24]. Whilst historically the heroin supply in Kenya may have been consolidated around a few suppliers, interviews with those involved in distribution demonstrate there has been a fragmentation of vertical chains of supply and emergence of small, ad hoc smuggling operations and new routes. In the word of Ndolo who used to sell heroin for Pakistani smugglers throughout the world until he finally settled down in his hometown Mombasa:

“I: So are there any international drug syndicates here?

R: No international syndicates. It’s just people. Right now, I have my own capital, 20,000. This is enough to buy 10 grams. I go to buy 10 grams, if I sell them, I keep the profit. I just have to look for someone to sell [for me], ‘Take this, sell it’ , ‘Take that, sell it’. That’s it. You hear a lot about ‘companies’, but they are not real companies.” (Ndolo, Mombasa)

Indeed, an increasing number of dealers are operating independently or working for a number of distribution operations. China, a respondent from Nairobi, used to work for one organisation as stock-keeper and ‘lab’ assistant processing heroin for 3 years. He subsequently started freelancing after disagreements emerged between him and his superior. In his words, chains of supply cut across distribution units, creating a networked drug supply, rather than strictly vertical hierarchies:

“R: This thing is like a chain, this is my stuff, then I have my subordinate and then it goes like that to the end. And the smallest guy…

I: So you think this chain is long?

R: It’s very long because, you see, for example, I have stuff and you know that I have stuff and you are also a dealer maybe. I will sell you some, and you probably have another person that sells to you. That’s how it goes, you can’t know.” (China, Nairobi)

The ‘chain’ that China refers to clearly has a vertical dimension to it but is also extended laterally as various freelancers and mediators connect different operations. As a result, top-down movements are interwoven with lateral ones; units are small, constantly regrouping and thus lack clear, long-term leadership.

This elongation and complication of the drug supply overlaps with disruption caused by policing strategies shaped with reference to Kenya and the region’s increasingly central position in the global War on Drugs and War on Terror [25, 26]. Recent policing initiatives reflect this. For example, the Mombasa county governor said on starting his term in office in 2013: ‘I today declare drugs a county-disaster and we are committed to fight it. Give me six months to deal with this drug menace’ [27]. Several chaotic, mass arrest operations in drug-dealing and using locations took place after this statement was made. The emphasis is on immediate arrests and deportations with the most visible actors in the heroin trade—those operating at the street level—being a target. In a renewed war on drugs initiative, following the first wave of counternarcotics activity in 2013, Mombasa county commissioner Nelson Marwa oversaw the raiding of 29 dealing locations in Mombasa county in September 2014. He reiterated a commitment to making as many arrests as possible, claiming that his team ‘will not spare anyone’ [28].

The Heroin Trade study documented a series of raids on one of the most prominent dealing locations on Mombasa Island, which took place in April 2013. Jay, former dealer and current stolen goods reseller from the locale, described the events as follows:

“After the campaign had finished, people got taken out of here by the police… They came about 4 pm, they put guards, dogs, they started arresting half of the people [break] many got arrested, few managed to run away . […] They burnt down the whole place, the police and the AP, so people can’t sleep here. Then they came back at 10 pm and then the following day too. About five times they came back. So people don’t sleep here anymore.” (Jay, Mombasa)

These unpredictable episodes of heavy counternarcotics policing introduce sudden shocks to local markets, drastically diminishing the availability of drugs. During such periods of intensified law enforcement, prices are likely to soar. In April 2013, for example, a single-dose sachet of heroin was sold for 300 Kenyan shillings—double its stable market price. Acting under international pressure, political leaders have taken bold steps to securitise the drug trade in the country. The highly publicised policing endeavours which are part of a national war on drugs [29], coupled with the fragmentation of supply networks, produce a highly fluctuating and unpredictable supply of heroin. As a female dealer from Mombasa concluded, ‘the quality of drugs changes every day’.

#### Managing the market

Out of this shifting, heavily policed drug supply and marginal position in the local economy, PWID experience acute economic pressures that need to be managed. That the drug does not give the same ‘kick’ or that it costs substantially more does not determine a transition, but in a context of economic vulnerability, withdrawals and social influence, the decision to inject is one way in which to cope with these shifts.

### The ‘feeling’ of injecting

A secondary narrative across accounts from the A2C study was of transitions to injecting focused on the ‘the feeling’ of injecting. This narrative was secondary across the data, figuring in 21 accounts. Whilst overlapping with a narrative of managing the market in that both focused on a ‘high’ and cost and quality factors figured, here we focus on how the search for ‘more steam’ was oriented towards the feeling of injecting. For example:

“I: Can you tell me how was it that you started injecting?

R: Starting injecting, I was seeing that when someone injected they were getting a lot of steam, they were feeling happy. I went and asked my friend why is it that when you inject you passing out so much? And she told why don’t I try … I told her fine let me try for today. I tried and felt the sweetness, which is how I went into it, just like that.

I: Mm, what can you say specifically made you inject?

R: Desire that is…your friend is feeling, desire

I: Okay that first time you were starting to inject, did you have any concerns about injecting?

R: No, I did not have any concerns, because I wanted to feel how someone feels when they injected. Because if it is smoking, I have smoked, if it is sniffing, I have sniffed, but how does someone feel when they inject?” (Mary, Ukunda)

Explication of this ‘feeling’ by PWID was limited; there were references to the high being ‘direct’ and making you ‘happy’, giving ‘pleasure’ and ‘taking you to the moon’ , whilst the feeling of smoking had subsided with increasing tolerance. Later, Mary references money, noting the money saved, but this figures as incidental to the dominant narrative of the ‘feeling’. Whilst cost and quality were sometimes mentioned, this was often absent from these accounts, suggesting a different pattern of influence from fluctuations in cost and quality, more indirect, if at all, and a greater emphasis on pleasure and curiosity.

As with an understanding of cost, the encouragement of friends and others injecting or wanting to replicate their experience was often central to accounts. Mary sought to inject based on observation of, and interaction with, a friend who was injecting. Tom talked of his sexual partner. He spoke about how he had started smoking ‘cocktails’ , and over time, they had stopped making him high. He then describes the day he first injected and how he was with a female friend:

“She told me try ‘Tom, this if you inject you will, you will fuck well, you will have intercourse, you feel pleasure, you and you will feel…you will enjoy a lot’. So it was my first time when she injected me, she was a doctor having a lot of experience and she is not a doctor for treating patients at the hospital” and later “but with that injection, it was something fantastic, the way I love steam, I see this is good.”

Such accounts of others reporting the pleasure and feeling of injecting and encouraging others were common. Tom also reported not being aware of the risk of HIV, but other accounts acknowledged risk, and it again figured as managed, with people buying or sourcing clean needles and syringes.

## Discussion

Transitions to injecting heroin in our study are described as multi-layered phenomena, emerging from a combination of factors to produce specific experiences. In our analysis, we have presented two narratives through which factors of deepening addiction, curiosity, the influence of friends, shifting cost and supply quality combine. Transitions are experienced as a process of managing a series of resource constraints or of curiosity or search for pleasure.

These narratives present differing experiences of the social and structural context and how risk for transitions is created. Particular features of the structural environment are experienced by some as constraints: a marginal position for PWID in the economy and particular shifts in the drug supply, themselves resulting from policing practices. Transitions as a social practice can therefore be seen to emerge from how agency is produced and enacted within these structural pressures through the creation of a logic of economic constraint that shapes how transitions are understood and managed [19]. Any choice to inject is then principally subsumed within an overall experience of resource constraint and economic vulnerability and figures to manage constraint rather than as an expression of freedom [30]. That these structural economic factors are not determining of transitions is then demonstrated by how economic vulnerability is secondary for some, as compared to a pursuit of pleasure. Here, a logic of pleasure emerges from the specific experience of a social network, whether through seeing others inject or being encouraged to inject. Our analysis therefore points towards the co-existence of differing effects of social and structural pressures, and so how risk is produced, based on the specificity of an individual’s experience and situation in relation to them.

These conclusions have implications for efforts to provide interventions in support of preventing transitions to injecting: of the need to respond to different experiences, and how, within this, there is a need to respond to structural as well as behavioural determinants of risk. For example, options other than a transition to injecting are needed as a strategy to manage long-term and acute poverty, such as livelihood support and more widespread drug treatment programmes. Responses to the poverty experienced by people who use drugs are also a priority to respond to how local economic cycles impact on PWID. The role of global and national economic policies and institutions in fostering local economic conditions should be a focus for further analysis with reference to responses to drug related harms (as well as their role in general economic uncertainty for the general population). Addressing poverty could include expansion of livelihood programmes as already in development in Kenya [31] and regulation and formalisation of tourist economies.

Interventions to address the fluctuating drug supply are limited by the nature of illegal organisations and the complex dynamics of policing them. However, this analysis does suggest that the current emphasis on criminalising low- and mid-level dealers, as well as users, has direct repercussions in leading to transitions, through creating an unreliable supply. In Kenya, anti-narcotics policing has recently centralised [32] increasing the likelihood of criminalisation of petty street-level dealers and, more frequently, heroin users, who are easy targets for regular police officers. Efforts towards supply-reduction could also cause longer term shifts in supply—as with the shift from brown sugar to white crest—with similar influences on modes of use. As such, emphasis should be placed on greater alignment at a strategic level between interdiction and health and social policy to ensure more emphasis on the latter to direct policing interventions that would minimise the harms of efforts to control supply. This could include a reduction in ‘low-level’ arrests and more focus on investigating networks as opposed to making ostentatious ‘small fish’ seizures and arrests. In addition, the development of clear emergency protocols to manage abrupt shifts in the drug supply is a priority [22].

For those whose experience is shaped by curiosity and pleasure, action on economic constraints or the drug supply may have less potential preventative impact. Instead, interventions that address the influence of a local social network may be of greater relevance [2]. In addition, engagement in outreach and ongoing counselling [33] could support the prevention of transitions or at minimum support them to be of less risk—i.e. a clean needle and syringe is available, with knowledge of safe use. A broader acceptance of the role of pleasure, and its incorporation into public health messaging and responses, is also essential [8].

A specific issue for further analysis is the increased vulnerability of women and the role of gender relations in creating risk for transitions. Research in other contexts has demonstrated women often transition in the context of intimate partnerships [9, 10]. Across our sample, women reported their transitions around the two narratives we have presented above. There were isolated references to boyfriends, but they were not described as integral to transitions. We did not discern any particular differences in transitions according to gender. Further probes within interviews to explore issues of gender in particular could have potentially identified further insight here. Additional analysis of how risk for transitions, and other drug-related harms, is gendered and is structured by gender relationships in this context is necessary to draw attention to the potential complexity around this issue [34].

This paper has sought to combine different datasets to give insight to the range of social and structural factors shaping transitions to injecting. Through this, we have added to the literature understanding transitions through qualitatively locating individual experiences in a detailed account of the heroin supply and surrounding economy and drawing attention to the role of pleasure.

Relating these datasets to each other is limited by how researchers hold different perspectives and interpretations, as well as familiarity with the specific contexts where data were collected. Nevertheless, ongoing discussion across the team to explore and understand the context for individual sections of data and explore our individual and then team-based interpretations helped overcome these challenges.

## Conclusions

In conclusion, transitions to injecting heroin in Kenya are shaped by specific features of the social context, drug trade and local economy. As harm reduction for people who inject drugs develops rapidly in Kenya, it is an urgent priority for HIV prevention to consider the needs of people who smoke drugs and to develop responses that can respond to the structural pressures that increase the risk of transitions.

## Abbreviations

HIV: human immunodeficiency virus
PWID: person or people who inject drugs

## References

[1] National AIDS Control Council of Kenya (2014). Kenya AIDS response progress report 2014, progress towards zero.

[2] Hunt N, Griffiths P, Southwell M, Stillwell G, Strang J (1999). Preventing and curtailing injecting drug use: a review of opportunities for developing and delivering route transition interventions. Drug Alcohol Review.

[3] Bravo MJ, Barrio G, Royuela L, Domingo L, Silva T (2003). Reasons for selecting an initial route of heroin administration and for subsequent transitions during a severe HIV epidemic. Addiction.

[4] Koram N, Liu H, Li J, Li J, Luo J, Nield J (2011). Role of social network dimensions in the transition to injection drug use: actions speak louder than words. AIDS Behav.

[5] Harocopos A, Goldsamt L, Kobrak P, Jost J, Clatts M (2009). New injectors and the social context of injection initiation. Int J Drug Policy.

[6] Kermode M, Longleng V, Singh B, Hocking J, Langkham B, Crofts N (2007). My first time: initiation into injecting drug use in Manipur and Nagaland, north-east India. Harm Reduct J.

[7] Kermode M, Longleng V, Singh BC, Bowen K, Rintoul A (2009). Killing time with enjoyment: a qualitative study of initiation into injecting drug use in north-east India. Subst Use Misuse.

[8] Fitzgerald JL, Louie R, Rosenthal D, Crofts N (1999). The meaning of the rush for initiates to injecting drug use. Contemp Drug Probl.

[9] Simmons J, Rajan S, McMahon JM (2012). Retrospective accounts of injection initiation in intimate partnerships. Int J Drug Policy.

[10] Goldsamt L, Harocopos A, Kobrak P, Jost J, Clatts M (2010). Circumstances, pedagogy and rationales for injection initiation among new drug injectors. J Community Health.

[11] Rhodes T, Singer M, Bourgois P, Friedman S, Strathdee S (2005). The social structural production of HIV risk among injecting drug users. Soc Sci Med.

[12] Beckerleg SE, Telfer M, Hundt GL (2005). The rise of injecting drug use in east Africa: a case study from Kenya. Harm Reduct J.

[13] Stimson GV (1998). Harm reduction in action: putting theory into practice. Int J Drug Policy.

[14] Harris M, Forseth K, Rhodes T (2015). “It’s Russian roulette”: adulteration, adverse effects and drug use transitions during the 2010/2011 United Kingdom heroin shortage. Int J Drug Policy.

[15] Rhodes T, Bivol S (2012). “Back then” and “nowadays”: social transition narratives in accounts of injecting drug use in an east European setting. Soc Sci Med.

[16] Rhodes T, Bivol S, Scutelniciuc O, Hunt N, Bernays S, Busza J (2011). Narrating the social relations of initiating injecting drug use: transitions in self and society. Int J Drug Policy.

[17] Werb D, Buxton J, Shoveller J, Richardson C, Rowell G, Wood E (2013). Interventions to prevent the initiation of injection drug use: a systematic review. Drug Alcohol Depend.

[18] Bridge J (2010). Route transition interventions: potential public health gains from reducing or preventing injecting. Int J Drug Policy.

[19] Rhodes T, Wagner K, Strathdee S, Shannon K, Davidson P, Bourgois P, O’Campo P, Dunn JR (2012). Structural violence and structural vulnerability within the risk environment: theoretical and methodological perspectives for a social epidemiology of HIV risk among injection drug users and sex workers. Rethinking social epidemiology, towards a science of change.

[20] Bourgois P (1998). The moral economies of homeless heroin addicts: confronting ethnography, HIV risk, and everyday violence in San Francisco shooting encampments. Subst Use Misuse.

[21] Deveau C, Levine B, Beckerleg SE (2006). Heroin use in Kenya and findings from a community based outreach programme to reduce the spread of HIV/AIDS. Afr J Drug Alcohol Stud.

[22] National AIDS & STI Control Programme (2014). Heroin crisis; its implications on the health of heroin users and government response.

[23] Ezzy D (2002). Qualitative analysis, practice and innovation.

[24] Wright J (2013). Transnational organised crime in eastern Africa: a threat assessment.

[25] Mayoyo P (2011). Kenya: Obama targets Mwau in drug war. The Nation (Nairobi).

[26] Obama B. Letter from the President on the Foreign Narcotics Kingpin Designation Act, June 1, 2011. www.whitehouse.gov/the-press-office/2011/06/01/letter-president-foreign-narcotics-kingpin-designation-act. Accessed 30 March 2015

[27] Joho fights drug problem. The Star, April 23, 2013.

[28] Mombasa county in renewed war on drugs. Capital News, 27 September 2014.

[29] Mutua Ndonga J (2014). Uhuru has proved he is walking the talk in the war against drugs. Daily Nation.

[30] Draus P, Roddy J, Kanzoni A (2015). Streets, strolls and spots: sex work, drug use and social space in Detroit. Int J Drug Policy.

[31] Ogembo HP, Angira CO, Mbugua B, Abdallah S, Abdool R. Reducing vulnerability of marginalized drug dependent communities in Nairobi Kenya through socioeconomic opportunities. 20th International AIDS Conference, Melbourne, Australia. 2014.

[32] Kimaiyo disbands anti-narcotics unit at the coast, shuts offices, standard digital, 10 November 2014. www.standardmedia.co.ke/m/story.php?articleID=2000140923&story_title=Kimaiyo-disbands-Anti-Narcotics-Unit-at-the-Coast-shuts-offices. Accessed 15 December 2014

[33] Needle RH, Burrows D, Friedman SR, Dorabjee J, Touzé G, Badrieva L, et al. Effectiveness of community-based outreach in preventing HIV/AIDS among injecting drug users. Int J Drug Policy. 2005;16:Supplement 1: 45–57.

[34] Syvertsen J, Robertson AM, Strathdee S, Martinez G, Rangel MG, Wagner K (2014). Rethinking risk: gender and injection drug-related HIV risk among female sex workers and their non-commercial partners along the Mexico-U.S. border. Int J Drug Policy.

